# Chronic Pulmonary Aspergillosis—Where Are We? and Where Are We Going?

**DOI:** 10.3390/jof2020018

**Published:** 2016-06-07

**Authors:** Gemma E. Hayes, Lilyann Novak-Frazer

**Affiliations:** 1 The University of Manchester, Oxford Road, Manchester M13 9PL, UK; lily.novak-frazer@manchester.ac.uk; 2 Manchester Academic Health Science Centre, 46 Grafton Street, Manchester M13 9NT, UK; 3 National Aspergillosis Centre, 2nd Floor Education and Research Centre, University Hospital of South Manchester, Southmoor Road, Manchester M23 9LT, UK; 4 The University of Manchester, Manchester Academic Health Science Centre, 2nd Floor Education and Research Centre, University Hospital of South Manchester, Southmoor Road, Manchester M23 9LT, UK; 5 Mycology Reference Centre, Manchester, 2nd Floor Education and Research Centre, University Hospital of South Manchester, Southmoor Road, Manchester M23 9LT, UK

**Keywords:** chronic pulmonary aspergillosis, chronic cavitary pulmonary aspergillosis, chronic fibrosing pulmonary aspergillosis, subacute invasive aspergillosis, aspergilloma, *Aspergillus* nodule, *Aspergillus*

## Abstract

Chronic pulmonary aspergillosis (CPA) is estimated to affect 3 million people worldwide making it an under recognised, but significant health problem across the globe, conferring significant morbidity and mortality. With variable disease forms, high levels of associated respiratory co-morbidity, limited therapeutic options and prolonged treatment strategies, CPA is a challenging disease for both patients and healthcare professionals. CPA can mimic smear-negative tuberculosis (TB), pulmonary histoplasmosis or coccidioidomycosis. Cultures for *Aspergillus* are usually negative, however, the detection of *Aspergillus* IgG is a simple and sensitive test widely used in diagnosis. When a fungal ball/aspergilloma is visible radiologically, the diagnosis has been made late. Sometimes weight loss and fatigue are predominant symptoms; pyrexia is rare. Despite the efforts of the mycology community, and significant strides being taken in optimising the care of these patients, much remains to be learnt about this patient population, the disease itself and the best use of available therapies, with the development of new therapies being a key priority. Here, current knowledge and practices are reviewed, and areas of research priority highlighted.

## 1. Introduction

The spectrum of disease caused by *Aspergillus* spp. is wide, with disease manifestations being governed by the underlying condition of the host immune system, which determines the nature of the host-*Aspergillus* interaction. The contribution of the host immune system is so great that the individual illnesses, collectively described as pulmonary aspergillosis, can be viewed as a continuous spectrum of disease, with evolution of disease entities occurring in association with changes in the host immune response [1]. Most commonly caused by *A. fumigatus*, chronic pulmonary aspergillosis (CPA) is seen in individuals with an ostensibly normal immune system and significant underlying lung disease. CPA affects an estimated 3 million people across the globe, with the disease conferring significant morbidity and mortality, making it a worldwide problem for healthcare economies [2,3,4]. Despite much progress being made in the recognition and treatment of this disease entity, much remains to be done in almost all areas associated with the disease. Here the current literature is reviewed and areas of research priority highlighted.

## 2. At Risk Individuals

CPA almost exclusively occurs in individuals with pre-existing cavitating or bullous lung disease with pulmonary tuberculosis, nontuberculous mycobacterial infection (NTM), allergic bronchopulmonary aspergillosis (ABPA), sarcoidosis, pneumothorax, chronic obstructive pulmonary disease (COPD), surgically treated lung cancer and bronchiectasis all placing patients at increased risk [5]. From a global point of view, previous pulmonary tuberculosis far outstrips the other causes as the leading predisposing factor for the development of CPA. Data from the most recent World Health Organisation Tuberculosis Report reports 6.3 million cases of notified TB in 2014 alone, with actual numbers likely to be a little higher due to failure of complete data capture [6]. The prevalence of CPA complicating TB is very variable and depends on local incidence rates of tuberculosis within the population. Initial estimates suggest that rates vary between <1 per 100,000 in the developed western nations and the USA with rates rising to 42.9 cases per 100,000 in the Democratic Republic of Congo and Nigeria [2]. Subsequent work has identified further countries where the burden of CPA is heavily driven by previous infection with pulmonary TB, including Vietnam, the Ukraine and Iran [7,8,9]. In areas where the incidence of TB is low, COPD appears to be the major risk factor for the development of CPA, with rates of between 33.3% and 42% reported [5,10,11,12,13]. These figures are based on the number of diagnosed cases, and given the high rates of under diagnosis the actual burden of disease is likely to be significantly higher.

The presence of current or previous NTM infection is equally as common as pulmonary tuberculosis in patients affected with CPA in countries where the incidence of *M. tuberculosis* is low [5]. In fact, recent research from the UK suggests that the incidence of NTM infection is rising [14]. Commonly involving infection with *Mycobacterium avium*, *xenopi*, *kansasii* or *malmoense*, NTM is reported in association with chronic cavitary, and subacute invasive, pulmonary aspergillosis and nodules may mimic, or co-exist, with *Aspergillus* nodules in nodular CPA [5,15,16,17,18]. Patients with bronchiectasis and NTM infection are at a higher risk of *Aspergillus* related lung disease, with higher levels of positive *Aspergillus* serology, when compared to patients without complicating NTM infection [19]. The associated co-infection seen likely represents susceptibility conferred by underlying pulmonary co-morbidity, although potential immune defects, such as a reduction in the production or response to γ interferon, could also contribute. Only cavitary disease and prolonged steroid use have been identified as independent risk factors for the development of CPA in patients with NTM [20].

Estimates suggest CPA complicates sarcoidosis in 3%–12% of cases, with an estimated global burden of 71,907 cases, predominantly distributed in the African subcontinent and Americas [21]. Disease is typically reported in those with severe fibrocavitary sarcoidosis, predominantly with an upper lobe distribution, with unilateral and bilateral disease described [22]. The outcome for this group of patients appears to be particularly poor, reflecting the severity of the underlying pulmonary fibrosis and the usual need for ongoing corticosteroid or second line immunosuppressive therapy [22,23,24]. 

The development of CPA following thoracic surgery for both malignant and non-malignant pathologies has been documented, with rates of 3.6% quoted when surgery was undertaken for malignancy, the incidence of CPA increasing with prolonged post-operative survival [5,25]. Factors influencing the development of CPA in this cohort include male gender, current smoking and co-existing COPD. Pulmonary malignancy treated with chemo/radiotherapy and radiofrequency ablation are independently linked to the development of aspergilloma and CPA [5,26,27,28].

### 2.1. Phenotypes in CPA

At present no study definitively demonstrates the presence of distinct disease phenotypes in CPA, a fact highlighted in the recent guidelines [29]. However the identification of individual disease phenotypes is attractive and would allow treatment plans and prognosis to be discussed on an individual patient basis. Current attempts at phenotyping in CPA have been limited, in part due to the multiple, and independent, factors contributing to the onset and development of disease [30]. Similarly, phenotyping is not straightforward as the majority of affected individuals have significant underlying respiratory co-morbidity, bringing multiple confounders to any analysis. Administration of inhaled or oral corticosteroids and other immunosuppressive agents is also common and further confounds interpretation. In COPD, where sputum positivity to *A. fumigatus* is associated with higher inhaled steroid dose and an increased risk of progression of CPA, it becomes harder to attribute a phenotype to CPA alone [31,32].

### 2.2. Immune Deficiency and CPA

Unlike invasive aspergillosis, CPA is not associated with an immunocompromised host; however a degree of mild immunosuppression may be present as a result of comorbid disease. Rarely, *de novo* cases are seen following HIV, chemotherapy and immunosuppressive or biological therapy, all of which have the potential to exacerbate pre-existing disease. However, for the most part, the immune system of these patients appears ostensibly normal, although subtle immune defects, many of which remain as yet unclassified, are increasingly recognised. Mannose binding lectin deficiency is one of these.

Mannose-binding lectin plays an important role in innate immunity. A reduction in functional levels of this protein has been demonstrated to predispose to invasive pulmonary aspergillosis in susceptible individuals [33]. This suggests that immunogenetic defects, as well as abnormalities in cellular immunity, may be important in conferring increased susceptibility to disease [34]. Impaired Th1 and Th17 immunity, with reduced production of both γ interferon and IL-12, have also been demonstrated, emphasising the importance of the adaptive immune response in host defence [35,36]. More recently the potential role of γ interferon in the pathogenesis of CPA has been explored further, suggesting that genetic or epigenetic factors involving the γ interferon gene may confer susceptibility to CPA [37].

### 2.3. Genetic Susceptibility and CPA

Individual genetic susceptibility to CPA is poorly understood although disease susceptibility is thought to be polygenic in nature. Single nucleotide point mutations (SNPs) in Toll-like receptor 1 (TLR-1), Dectin-1, PLAT (plasminogen activator tissue), VEGFA (vascular endothelial growth factor A), DENND1B (DENN/MADD Domain Containing 1B), IL-1β, IL-1RN and IL-15 genes all confer putative susceptibility to CPA following genetic analysis of affected individuals and further work remains ongoing [38,39]. These SNPs represent in part a reduced ability of the human host to recognise and switch on appropriate defence responses, resulting in persistent inflammation, and subsequently fibrosis.

Differential gene expression and alteration of regulatory pathways may also prove to be as important as mutations and SNPs within individual genes. Using monocyte-derived macrophages from affected individuals, a 27.7 fold increase was seen in expression of the gene encoding pro-platelet basic protein (PPBP), a powerful neutrophil chemoattractant, with an associated increase in secreted PPBP [40]. However no SNPs were found to be significantly associated with the development of CPA. This suggests that an over-exuberant host response, perhaps due to a loss of regulatory pathways or in response to the surrounding inflammatory milieu, may be more important than any one individual mutation, predisposing individuals to persistent inflammation and parenchymal damage.

More work is needed to establish the true genetic and immune basis of CPA and it is expected exome sequencing of affected individuals will demonstrate the polygenic nature of genetic susceptibility, with multiple and varied mutations demonstrated within each individual tested. The challenge thereafter will be to elicit the significance of individual mutations at both a cellular and patient level, facilitating the development of meaningful diagnostic and therapeutic interventions that can be applied to both individuals and populations.

## 3. Clinical Manifestations

**Figure 1:**
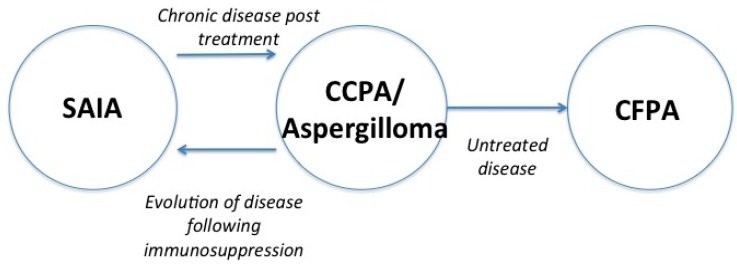
Forms of chronic pulmonary aspergillosis (SAIA: subacute invasive aspergillosis, CCPA: chronic cavitary pulmonary aspergillosis, CFPA: chronic fibrosing pulmonary aspergillosis).

The term CPA is most commonly used to describe the terms chronic cavitary and chronic fibrosing pulmonary aspergillosis (CCPA and CFPA respectively) and simple aspergilloma; although simple aspergilloma may be considered a separate entity in terms of both clinical and radiological presentation and management. Subacute invasive aspergillosis (SAIA), previously known as chronic necrotizing pulmonary aspergillosis (CNPA) or semi-invasive aspergillosis, and *Aspergillus* nodules are often also included in this group. Individual conditions are not mutually discrete and evolution between disease forms can be seen in the presence or absence of antifungal treatment or alterations in the state of the host immune system (Figure 1).

However these disease descriptions have been challenged, on the grounds that the subtype classification is over simplistic, difficult to apply clinically and of only academic interest [41,42,43]. It can be argued that the continuum of radiological and clinical features demonstrated within this population, combined with largely shared management principles, place the individual disease subtypes on a flexible, and potentially changeable, spectrum. Recent analysis using a subject centred, multivariate clustering approach, of a large series of patients with CPA, excluding those with single aspergilloma, demonstrated a high degree of homology with only one phenotype identified on cluster analysis [43]. Neither CCPA or CNPA were identified as individual clusters, confirming the findings of Izumikawa *et al.* who highlighted the potential difficulty of identifying these subtypes based on clinical and radiological findings alone [44,45]. Given the shared, and homogenous, characteristics of patients with CPA a new term “chronic progressive pulmonary aspergillosis”, encompassing all current disease subtypes, has been proposed, which is a useful clinical indicator for active therapy [45].

### 3.1. Aspergilloma

Forming the most recognised form of CPA, an aspergilloma represents a solid mass of *Aspergillus* hyphae, fibrin, mucus and other cellular debris, formed within a pre-existing area of pulmonary scar or cavity [10,29,31,46]. Aspergilloma can occur in isolation, where they are termed “single pulmonary aspergillomas”, or may co-exist in the context of either CCPA or CFPA.

The term single (or simple) pulmonary aspergilloma describes a single fungal ball in a single pulmonary cavity in the absence of any other signs of CCPA and usually describes those found after infection with *M. tuberculosis* [5,47,48]. In contrast with other forms of CPA, simple aspergillomas run an indolent course and are only very slowly progressive, with many being detected incidentally [47,49]. Should symptoms occur, the most commonly reported feature is haemoptysis, arising from stimulation of additional surrounding bronchial vasculature. Given the more “benign course” of simple aspergilloma, there has been some debate about its’ inclusion as a subgroup of CPA [50]. However given the close association between aspergilloma and CCPA/CFPA it remains under the umbrella of CPA at present [29].

### 3.2. Chronic Cavitary Pulmonary Aspergillosis (CCPA)

**Figure 2:**
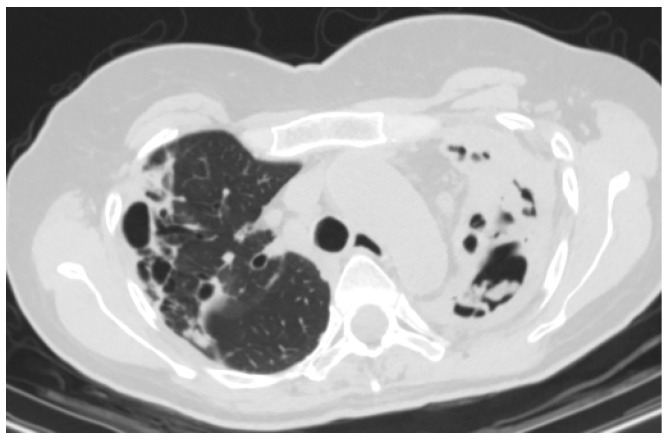
CCPA with involvement of the left upper lobe.

CCPA describes a combination of radiological, respiratory and systemic symptoms present for at least three months secondary to infection with *A. fumigatus* on a background of pre-existing chronic lung disease [29]. The pulmonary and systemic symptoms reflect a possible granulomatous or chronic inflammatory reaction leading to pleural thickening and parenchymal necrosis resulting in parenchymal destruction and the formation of predominantly thick walled cavities, the radiological hall mark of the condition (Figure 2) [47]. Up to half of these cavities may contain an aspergilloma [47]. The natural history of untreated CCPA can be described as progressively enlarging and coalescing cavities with developing pericavitary infiltrates/consolidation and potential pleural spread. Ultimately, fibrosis of the diseased segments occurs leading to the development of CFPA [10,29].

### 3.3. Chronic Fibrosing Pulmonary Aspergillosis (CFPA)

**Figure 3:**
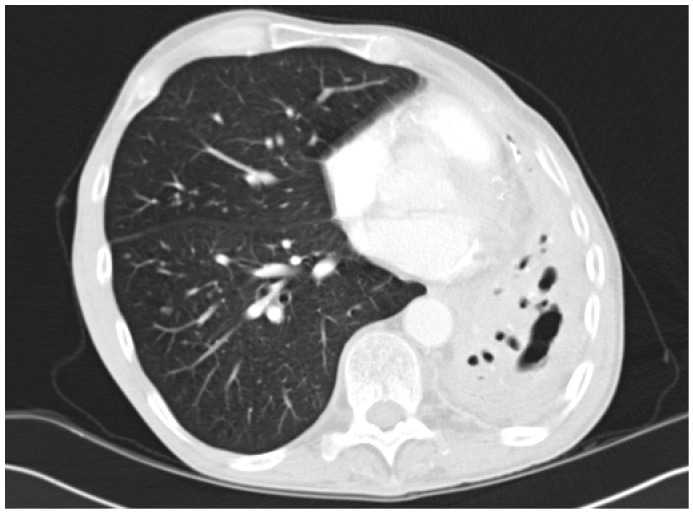
CT scan demonstrating CFPA with fibrosis and cavitation of the left lung resulting in significant volume loss in the left hemi-thorax.

**Figure 4:**
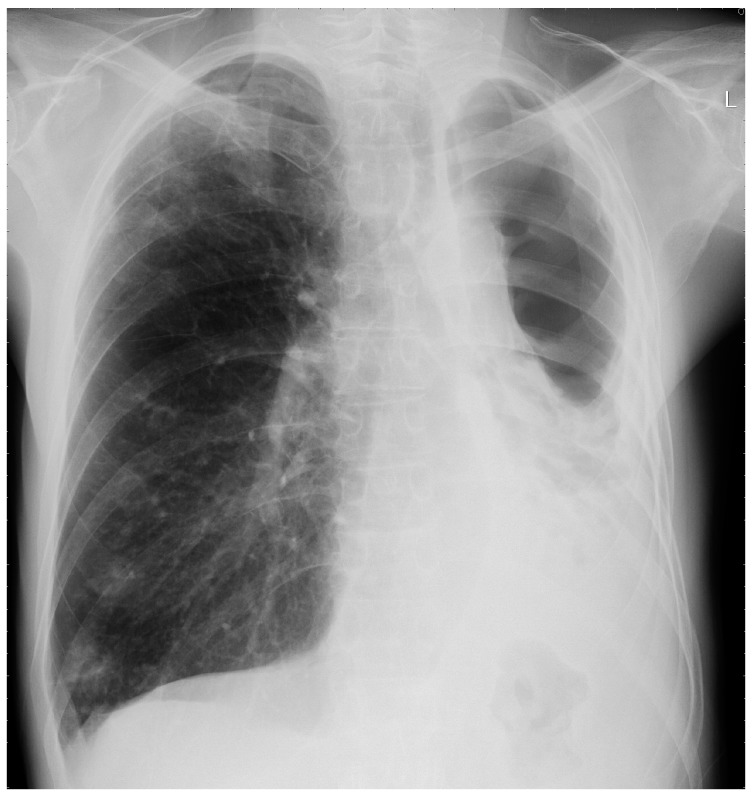
CFPA of the left lung with associated cavitation and volume loss.

Most commonly arising from untreated CCPA, and occasionally SAIA, CFPA is a disabling condition usually conferring significant respiratory embarrassment [10]. Extensive fibrotic destruction is usually seen affecting at least two lobes of the lung, often the whole hemi-thorax, and is sadly irreversible (Figure 3 and Figure 4) [29]. Although evolving from CCPA, aspergillomas are rare in the cavities associated with CFPA [10].

### 3.4. Subacute Invasive Aspergillosis (SAIA)

Sharing characteristics with both invasive and CCPA, SAIA is a rapidly progressive manifestation of pulmonary aspergillosis occurring in those who have a degree of immunocompromise or are profoundly debilitated [29,47] (Table 1). Symptoms progress over a period of weeks rather than months making prompt diagnosis and treatment of paramount importance.

### 3.5. Aspergillus nodules

**Figure 5:**
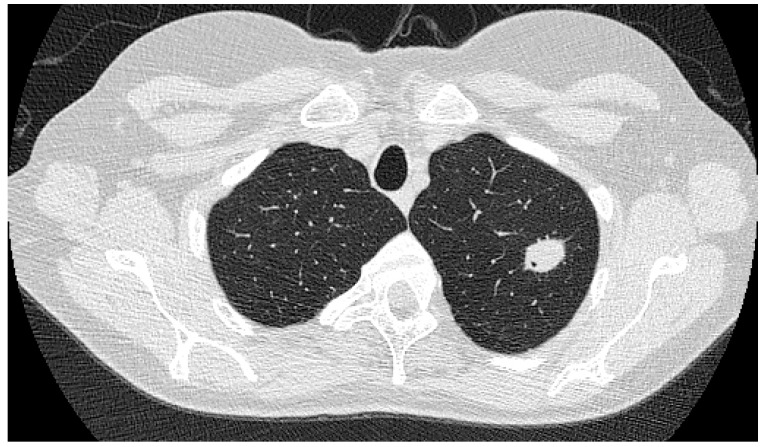
*Aspergillus* nodule—CT scan from a patient with an isolated pulmonary nodule attributable to *Aspergillus* spp., unusually this lesion is showing signs of early cavitation.

*Aspergillus* nodules are an uncommon form of CPA. Existing as either solitary or multiple lesions, diagnosis is almost always made after excision biopsy where histology confirms the presence of fungal hyphae without the presence of tissue invasion. The majority of *Aspergillus* nodules are less than 3 cm in diameter and do not cavitate, appearing as solid lesions on imaging (Figure 5). Often diagnosed incidentally the differential diagnosis for an *Aspergillus* nodule includes lung cancer, pulmonary metastases, cryptococcal nodules, coccidioidomycosis, nontuberculous and tuberculous mycobacteria and other rare pathogens [15,29,31,55,56,57,58]. Rheumatoid nodules may also mimic *Aspergillus* nodules and both lesions can co-exist in the same patient [29,59]. Rarely, mass lesions secondary to *Aspergillus* are seen. These are characterised by having a diameter >3 cm and demonstrate areas of central necrosis [29].

The need for excision biopsy can prove problematic in this cohort of patients as associated multi-morbidity may preclude surgical intervention. Radial endobronchial ultrasound (EBUS) provides an attractive alternative in this setting. With the ability to localize peripheral pulmonary nodules, including those <2 cm in diameter, and facilitate biopsy, EBUS diagnostic success rates of between 58%–88% have been reported, with success dependent on lesion size. [60,61,62,63]. Radial endobronchial ultrasound is therefore likely to have an increasing role in the diagnosis of patients with solitary or multiple nodules thought to be secondary to *Aspergillus* spp.

## 4. Diagnosis and Diagnostic Barriers

Given the complexities surrounding the diagnosis and identification of the multiple disease forms that make up CPA, recent efforts have focused on the development of standardised diagnostic criteria [5,47,50]. These efforts have been condensed into the recently published European guidelines “Chronic pulmonary aspergillosis—rational and clinical guidelines for diagnosis and management” [29]. These guidelines define CPA as follows “The appearance of either symptoms or radiological abnormalities for a period of greater than 3 months in the absence of significant immunosuppression with serological, immunological or microbial evidence of *Aspergillus* infection, and the exclusion of an alternative diagnosis or the recognition of an underlying concomitant respiratory condition”.

Each aspect of the diagnostic criteria is discussed in more detail below and key investigations are highlighted in Table 2.

### 4.1. Symptoms

The symptoms and signs of CPA, particularly CFPA and CCPA, are insidious, may often be masked by multiple existing respiratory morbidities, and can be split into respiratory and constitutional upset. Respiratory symptoms include ongoing productive cough, breathlessness and chest pain, with the onset of haemoptysis heralding the development of an aspergilloma [31,47,49]. Constitutionally, fevers, or swinging fevers, are often absent with weight loss, malaise, poor appetite and sweats predominating. None of these symptoms are pathognomic for CPA and the differential diagnosis is often wide with both malignancy and pulmonary tuberculosis featuring heavily (Table 3). Given the respiratory multi-morbidity associated with CPA it is not uncommon to find many of these conditions co-existing with CPA.

Although the symptoms described above are common, it is important to note that both single aspergilloma and *Aspergillus* nodules may be entirely asymptomatic, with diagnosis being incidental following routine radiology.

### 4.2. Radiology

The radiological changes associated with CPA are derived of changes related to each of underlying lung disease, long term inflammation secondary to chronic infection and the direct impact of *Aspergillus* [49]. The mainstay of imaging in CPA worldwide is the chest X-ray, although it is undoubtedly the case that CT scans identify location, distribution and extent of disease with a much greater degree of definition [29].

**Figure 6:**
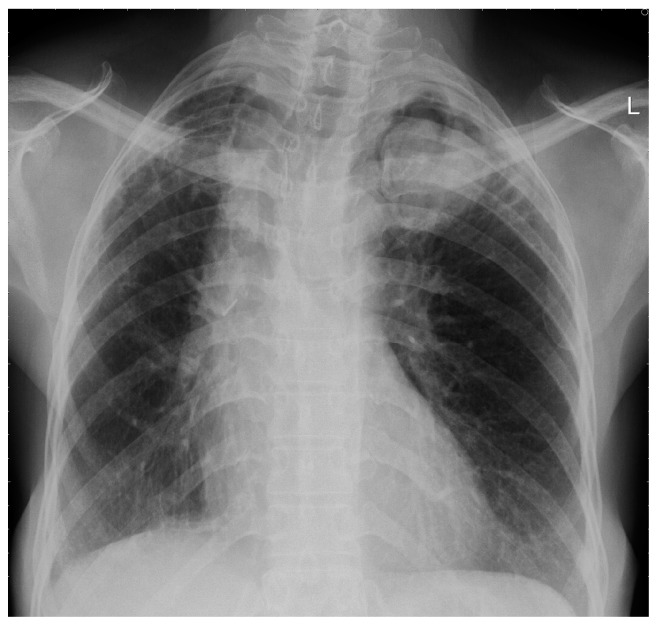
Solitary aspergilloma—Chest X-ray (CXR) demonstrating a left upper lobe aspergilloma in a patient with sarcoidosis.

The most well recognised radiological feature of CPA is the presence of an aspergilloma within an existing cavity (Figure 6), often heralded by prior thickening and irregularity of the cavity wall in which it sits [49,64]. Often found in the upper lobes, aspergillomas can be either solid mass like lesions or consist of a latticework of fungal strands containing air spaces within [29,64,65,66]. For patients with single aspergilloma a solid mass like lesion is the only radiological sign present which can lead to diagnostic uncertainty and delay in diagnosis [67]. For peripheral lesions, associated thickening of the adjacent pleura is also seen [66]. Although solitary aspergilloma are widely recognised it is not unusual for multiple aspergilloma to be present in either unilateral or bilateral distribution. The presence of an aspergilloma is not necessary for the diagnosis of CPA, with up to half of cavities present in CPA not containing fungal balls [47].

The key radiological features of CCPA are thick walled, slowly expanding cavities surrounded by areas of dense consolidation with associated pleural thickening, abnormal enhancement of extrapleural fat and a degree of parenchymal destruction [10,31,68]. Complex pleuro-parenchymal change, including *Aspergillus* empyema, should spores spread, or rupture into the pleural space is also occasionally seen [47,49]. All of the above are slowly progressive, with enlargement and coalescence of cavities over time, again with unilateral and bilateral disease documented. 

CFPA manifests as dense fibrotic change on both CT scans and X-rays and only the presence of adjacent cavitation or associated aspergilloma provide clues as to the underlying aetiology. Significant distortion of the associated structures within the hemi-thorax is also seen, as is bronchiectasis associated with both traction and chronic infection [10,47]. 

**Figure 7:**
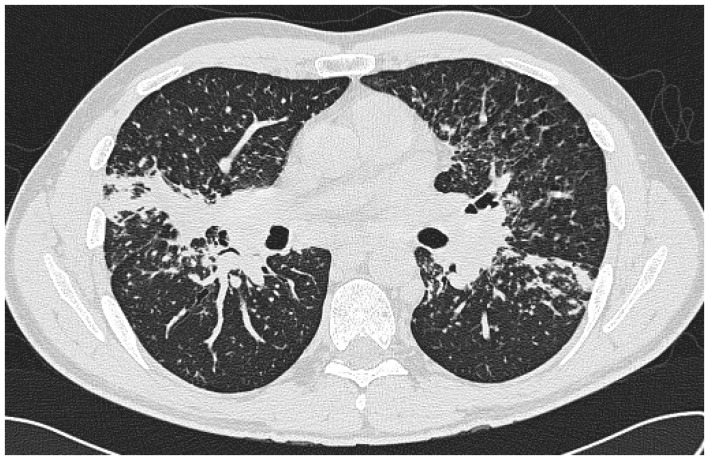
CT scan of SAIA with widespread consolidation.

**Figure 8:**
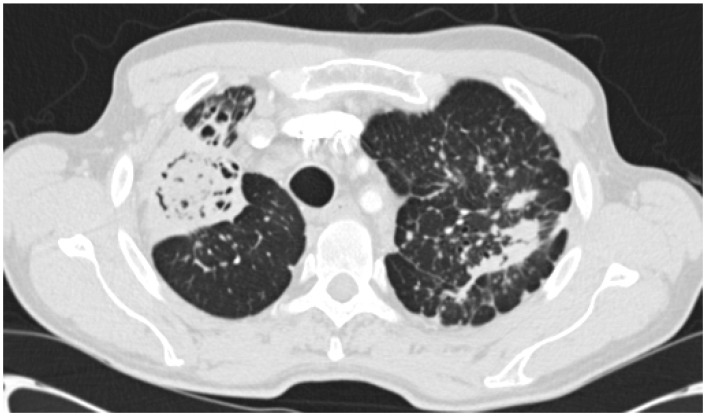
CT scan of SAIA demonstrating right upper lobe aspergilloma with associated pleural thickening and left upper lobe consolidation.

The radiological changes associated with SAIA differ from those seen in CCPA in that pre-existing cavitation is unusual [66,68,69,70]. Changes can be summarised as progressive upper lobe consolidation with cavitation leading to a rapidly expanding thin walled cavity (Figure 7). Worsening parenchymal necrosis can lead to the development of an air crescent sign and pleural thickening, effusion, pneumothorax and a fungal ball can also be seen (Figure 8) [50,69,71].

**Figure 9:**
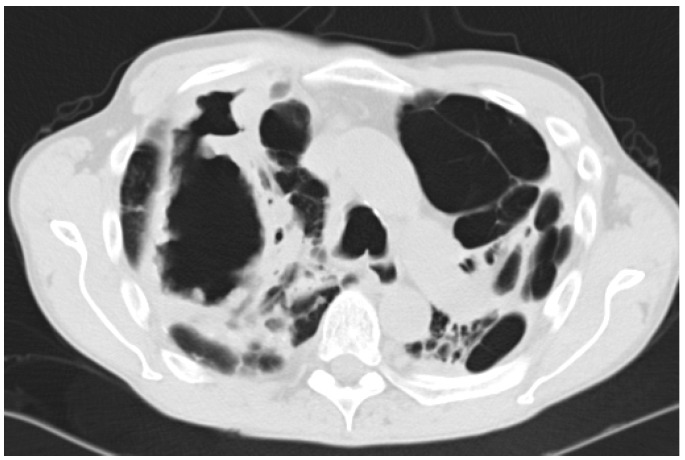
Identification of co-existent pathologies: CT scan from a patient with CCPA, *M. avium intracellulare* and severe bullous emphysema.

The radiological features described above are not all characteristic of CPA and radiology alone is often unable to confirm diagnosis. Moreover changes are often assigned to other, more common, pathologies inevitably leading to delay and disease progression. Common pathologies may also co-exist with CPA, further complicating the assignment of causality to radiological features (Figure 9). Until recently, no radiological criteria, which either classified or monitored progression of disease, were published and the use of other imaging modalities, for example positron emission tomography (PET)-CT, in the diagnosis of CPA had so far failed [67,72]. The use of composite radiological endpoints have however been explored by various investigators as a marker of treatment success. The absence of standardised and objective radiological criteria prevented direct comparison between studies [73,74,75]. The recent analyses of defined and objective radiological features associated with response to treatment with antifungal therapy authored by Godet *et al.* is therefore welcomed [76].

Godet *et al.* undertook a retrospective review of cross sectional thoracic CT imaging in 36 patients with CPA at both baseline and 6 months following appropriate antifungal therapy. A statistically significant association between reduction in cavity wall and/or pleural thickness and improved clinical condition was identified. Similarly a strong, but non-significant, association between disappearance of a previously identified fungal ball and a reduction in cavity wall and/or pleural thickness and clinical improvement was also demonstrated. In contrast, the evolution of either *Aspergillus* nodules or cavities correlated poorly with clinical course [76]. These findings allow, for the first time, objective radiological criteria to be employed in clinical practice to assess therapeutic response to treatment and facilitate long term monitoring. Indeed current guidelines now recommend repeat low dose CT for all patients at three or six months following initiation of treatment to assess response [29]. These findings are also valuable to the research community, providing a means of assessing response to current and novel antifungal therapy in future studies. 

Wider recognition of *Aspergillus* as a potential cause for common radiological abnormalities and standardised radiological reporting system are required to adequately monitor disease progression and the impact of anti-fungal therapy. On a global scale, increased access to cross-sectional imaging will allow clinicians to identify more readily affected individuals across the developing world and administer effective and appropriate treatment.

### 4.3. Serology and Immunology

If CPA is suspected, an *Aspergillus* IgG test is essential to confirm *Aspergillus* infection which, in combination with the presence of a fungal ball, will be positive in >90% of cases [29]. Moreover, a positive *Aspergillus* IgG also has a positive predictive value of 100% in differentiating infected and colonized individuals, making it a powerful diagnostic tool when used in combination with other diagnostic modalities [77]. A positive *Aspergillus* IgG is however not unique to the diagnosis of CPA and can be found in a variety of other conditions (Table 4). Despite this, the use of *Aspergillus* IgG antibody as a diagnostic tool is far superior to the use of *Aspergillus* precipitins, which are demonstrably less sensitive than the available automated *Aspergillus* IgG antibody assays, and should always be used as the gold standard, where available, to confirm infection [78,79,80,81,82,83].

The *Aspergillus* antibody response, its role in the diagnosis and management of CPA, and a summary of the various techniques available for monitoring treatment, have been summarised recently [84]. Key points from this body of evidence include confirmation that an elevated *Aspergillus* specific IgG is more sensitive than IgA, M or E in the diagnosis of CPA, there is a role for measurement of IgG beyond diagnosis and that although multiple laboratory methods are used for testing *Aspergillus* IgG, little is known about comparative efficacy of methodology [48,73,74,84,85]. The use of multiple methods to provide serological confirmation of *Aspergillus* infection is a cause for concern and it is not clear that the performance of these tests is comparable between hospitals or individual patients groups within the CPA population. Standardisation of techniques, re-evaluation of controls and, perhaps, reassignment of normal levels, is needed to ensure that antibody testing remains relevant and appropriate to the target population [80].

It should also be noted that in a small group of patients with CPA, the *Aspergillus* IgG may remain negative even in the presence of symptoms, radiology and laboratory diagnostics suggestive of disease [29,84]. Some tests perform better than others [80]. The reasons for this are unclear but may include hypogammaglobulinaemia, failure to mount an appropriate antibody response to *Aspergillus* or infection with a species other than *A. fumigatus*. It is for this reason that the absence of *Aspergillus* IgG or precipitins cannot be used as a definitive tool to exclude the diagnosis. A negative *Aspergillus* IgG test may be seen in the presence of a single, stable aspergilloma or *Aspergillus* nodules [29,84]. In cases where antibody testing is inconclusive but CPA is suspected (and differential diagnoses, including allergic forms of aspergillosis, have been eliminated), evidence to support diagnosis should be sought using alternative techniques.

Sensitisation to *Aspergillus* spp. is often seen in patients with CPA and total IgE and *Aspergillus fumigatus*-specific IgE levels are often, although not consistently, elevated [10,50]. This is most commonly seen in patients with asthma and ABPA which has progressed to CPA or in those with cystic fibrosis [1,29].

As well as the serological changes described above, CPA is often associated with chronic elevation of serum markers of systemic inflammation including C-reactive protein (CRP), plasma viscosity (PV) and/or erythrocyte sedimentation rate (ESR) [10]. A polyclonal rise in immunoglobulins following gel electrophoresis is also often seen. None of these markers are specific to CPA and elevation may be difficult to interpret in the context of chronic disease. Further increases may not represent disease progression, rather superadded bacterial infection or antecedent illness. 

### 4.4. Histopathology

Histologically, CPA is characterised by the absence of invasion of either pulmonary parenchyma or vasculature [11,47,49]. The presence of vascular and parenchymal invasion by hyphae, in combination with an acute inflammatory exudate or necrosis, is not compatible with a diagnosis of CCPA, and raises the suspicion of SAIA or invasive disease [29,86]. Histology from patients with CCPA commonly demonstrates chronic inflammatory change, although rarely with granulomata, with fungal hyphae contained within cavities [49,87]. 

Histopathological evaluation of biopsied material confers significant benefits over culture or direct microscopy alone, with direct tissue staining often identifying the fungus involved and establishing whether there is infection, colonisation or contamination of the sampled tissue [88,89]. However this technique is also limited and identification of fungi present based on size and morphological characteristics, such as septate, narrow-angle-branching hyphae, can be nonspecific.

### 4.5. Culture of Respiratory Secretions

The presence of *Aspergillus* spp. in either sputum or bronchoalveolar lavage fluid following cultures supports, but does not confirm, a diagnosis of CPA given that *A. fumigatus* is a ubiquitous pathogen, can be present in the context of multiple other respiratory conditions and may contaminate laboratory cultures [29]. Low rates of culture positivity, which fall further in the presence of treated disease, also limit the use of culture of respiratory tract secretions as a diagnostic tool [10,11,90]. Similarly, negative cultures do not rule out a diagnosis when clinical suspicion is high and supported by radiological and serological data [91]. 

Concerns surrounding the methodology used for culture of respiratory secretions for use in the diagnosis of CPA, specifically the low yield of *Aspergillus* spp. when using standard processing procedures, have also limited the utility of this technique. These typically underestimate the presence and amount of filamentous fungi, including *Aspergillus*, present from patients who are known to suffer from pulmonary aspergillosis [92]. However, newer techniques involving the inoculation of higher volumes of undiluted sputum, so called “high volume fungal culture” and the use of fungal specific media have partially alleviated this problem [93,94]. This makes the use of sputum culture a particularly useful adjunctive tool in the diagnosis of CPA, given that bronchoscopy is often precluded by respiratory multi-morbidity. However the presence of *A. fumigatus* on bronchoalveolar lavage fluid is much more common in the presence of infection, when compared to colonisation, which makes these samples more sensitive and specific if they can be obtained [77]. 

The importance of culture of respiratory tract secretions extends beyond diagnosis as a persistently positive culture despite adequate antifungal therapy suggests the development of a resistant strain of *Aspergillus* [95].

### 4.6. PCR of Respiratory Secretions

The appeal of using molecular detection methods to diagnose and track CPA exacerbations is their much higher sensitivity over culture [84,93,96,97,98,99,100]. However, although PCR on respiratory samples has been demonstrated to have a sensitivity of 77% in patients with cystic fibrosis, it has never been formally evaluated in the context of CPA [101]. Thus PCR for *Aspergillus* spp. on respiratory samples can only support rather than confirm diagnosis [29]. 

Despite this the utility of respiratory sample PCR for *Aspergillus* spp. within the CPA population is likely to be high as sputum sampling can be undertaken frequently and easily with little difficulty. Moreover strong signal strength correlates well with pulmonary infection and serial values can be used to track the success or failure of treatment in a non-invasive manner. PCR also shows promise in identifying the development of anti-fungal resistance, either through the identification of strongly positive PCR samples on treatment or by directly detecting resistance mechanisms [96].

However the institution of respiratory sample PCR into widespread clinical use will require standardisation of the technique across mycology laboratories with an international consensus agreement on levels representative of colonisation, infection and exacerbation or the development of resistance. Reports of the sensitivities and specificities of commercially available and in-house *Aspergillus* PCR detection kits from different clinical trials using respiratory and non-respiratory specimens currently vary considerably due to the breadth of targets (predominantly but not limited to the rRNA genes of *Aspergillus* spp.), the choice of primers, the method for identification of amplified DNA and the method for DNA isolation prior to the amplification process [102]. All of these will need to be addressed before widespread implementation when it is likely that PCR will form part of a cluster of diagnostic criteria, as is the case with invasive disease, rather than a gold standard diagnostic criterion [103].

### 4.7. Galactomannan Assay of Respiratory Secretions and Serum

Galactomannan (GM) is a component polysaccharide of the *Aspergillus* spp. cell wall and is released into the surrounding host environment during active fungal growth or tissue invasion [104]. Whilst the new ESCMID/ERS guidelines for the diagnosis and management of CPA endorse the use of galactomannan as a diagnostic aid, the majority of evidence for diagnostic use comes from studies of invasive disease and diagnostic utility in CPA remains controversial [29,103,105,106,107]. As an assay, GM also demonstrates significant flaws and a degree of cross-reactivity with other fungi including *Penicillium* and *Histoplasma* spp. [108]. Concurrent treatment with β-lactam antibiotics, for example piperacillin-tazobactam, leads to false positive results and antifungal therapy significantly lowers assay sensitivity [109,110]. The use of both these agents is widespread within the CPA population, significantly limiting diagnostic utility. Additionally, the sensitivity of GM testing is significantly reduced in the presence of *Aspergillus* specific antibodies, putatively due to direct binding of these antibodies to GM antigen.

Despite these problems, identification of GM in respiratory secretions, predominantly bronchoalveolar lavage (BAL) samples, has been demonstrated to be more effective than serum GM in determining the presence of *Aspergillus* spp. in the CPA population [111]. Sensitivities of 85.7% and 92% have been reported in patients with aspergilloma and SAIA, respectively, using an optical density index (ODI) of 0.5, or greater, as the threshold of positivity [106,112]. Sensitivity in this context is much higher in BAL samples when compared to serum; however, specificity differs little with figures of 76.3 *vs.* 78.9% quoted [112]. 

Few studies support the effectiveness of GM testing of serum samples in this patient group due to low sensitivity. Recent work has demonstrated serum GM antigen positivity in 23% of patients with CPA and in 15% of patients without, resulting in positive and negative predictive values outside those necessary for wide spread clinical implementation [113]. Further work has also demonstrated inferiority of serum GM testing when compared to *Aspergillus* precipitating antibody tests [114]. The level of GM serum positivity in this patient group is also low, suggesting that serum GM should not be used as a primary diagnostic tool [48]. This contrasts sharply with invasive aspergillosis where GM can be a highly sensitive diagnostic tool. It is therefore unsurprising that serum GM positivity increases in patients with SAIA where the likelihood of vascular invasion is higher [106,112,115,116]. Serum GM may therefore be a useful marker for both diagnosis and monitoring in this small subgroup of patients. 

Given the current paucity of evidence surrounding the use of GM in the diagnosis and monitoring of CPA, and the inherent difficulties in obtaining BAL samples on a routine basis, the GM assay can only be used as a supporting diagnostic tool unless the radiological appearance is classical and the GM assay strongly positive [29]. Assessment of the utility of sputum GM measurement is an area of active research interest due to the ease in which samples can be produced and processed in a wide variety of clinical settings. 

### 4.8. New and Emerging Technologies

#### 4.8.1. MALDI-TOF Mass Spectrometry in the Detection of *Aspergillus* spp.

The integral importance of fungal culture in the identification of *Aspergillus* spp. from patient specimens is being utilised in the development of MALDI-TOF mass spectrometry for identification of cultured fungi. Already well established in the routine identification of clinically important pathogenic bacteria and yeasts, this technique uses mass spectrometry to create individual protein spectra from patient cultures, which can then be matched to a reference database [117]. Not yet utilised widely by regional mycology laboratories, developments in sample preparation protocols and specific fungal databases for identification have highlighted the utility of this tool for the identification of *Aspergillus* [118,119,120]. Discrimination of morphologically and phylogenetically similar species of *Aspergillus* has been achieved using this technique despite their significant biodiversity [121,122].

The introduction of MALDI-TOF MS-based identification for *Aspergillus spp.*, and other filamentous fungi, within routine clinical practice requires development of existing experimental methodology. Confirmation of correlation between taxonomy and the spectral patterns produced is critical before this technique achieves a place in clinical practice, and reference databases of both pathological strains and their associated patterns need to be established [123]. Moreover, established and successful individual laboratory methods need to be developed, with large multicentre studies required to standardise a MALDI-TOF MS-based mould identification procedure [121].

#### 4.8.2. The Future

Although not yet explored in this patient cohort, much interest has focused around the development of a lateral-flow device (LFD) test for use in invasive aspergillosis [124]. Simple, inexpensive and providing rapid diagnosis at the point of care (POC), a similar antibody-based test for CPA would revolutionise diagnosis and have a profound impact as a screening tool in resource poor settings where incidence is high but access to diagnostics and healthcare low. A similar device that detected *Aspergillus* antigen, rather than antibody, would also play a key role in early diagnostics, identifying the presence of *Aspergillus* prior to the availability of cultured specimens. 

Similarly, interest in the use of both “electronic nose” and gas chromatography-mass spectrometry technology to assess exhaled volatile organic compounds in respiratory diseases including asthma, COPD and pulmonary aspergillosis is also growing. Again this may prove useful as a screening tool but is likely to be limited by multiple environmental confounders and access to technology in the immediate future [89,125,126,127,128,129,130,131].

## 5. Treatment Options

Treatment in CPA aims to alleviate symptom burden for affected individuals, reduce episodes of haemoptysis and prevent pulmonary fibrosis, thus preserving lung function. This approach often takes three forms, control of *Aspergillus*, control of complications and control of co-existent co-morbidities; thus treatment is often a lifelong undertaking. The evidence base for specific treatment interventions in this patient population is limited by the small number of patients affected by the condition and the impact this has on the generation of robust randomised control trials. However, as discussed above guidelines, largely based on expert opinion, do exist [29,132]. The implementation of these guidelines requires the presence of a large multidisciplinary team including thoracic medicine, infectious diseases and specialist respiratory nursing expertise, thoracic surgeons, respiratory physiotherapy, dietetics input and palliative care. This facilitates rapid and expert treatment of comorbidities, the provision of both nutritional support and pulmonary rehabilitation and the development of individual treatment programmes [47].

### 5.1. Current Antifungal Agents—Azoles, Echinocandins and Liposomal Amphotericin B

#### 5.1.1. Triazole Therapy

Triazole therapy forms the cornerstone of oral treatment for CPA and treatment is now considered standard care [29]. Acting through inhibition of fungal CYP51p, triazole therapy inhibits the conversion of lanosterol to ergosterol, disrupting the structure and function of the fungal cell membrane thus exerting fungistatic, and in certain circumstances, fungicidal effects on *Aspergillus*. The spectrum of antifungal activity of itraconazole, voriconazole and posaconazole encompasses *Aspergillus* spp. and all three agents are used sequentially in the treatment of CPA according to clinical need. Few studies exist on the efficacy of azole therapy in the context of CPA and certainly itraconazole remains the first line drug of choice for the management of patients with CPA. An important exception to this is the management of single aspergilloma where, if co-morbidities permit, definitive management takes the form of surgical resection, which provides the best chance of long term cure [29,133,134,135].

Evidence for the use of itraconazole is supported by a small randomised control trial and data from case series [10,75,136,137,138]. Comparing itraconazole to supportive care over a six month period, a composite endpoint encompassing clinical and radiological features suggested an overall response rate of 76.5% [75]. Recent data has suggested that a weight-based, variable dosing schedule for itraconazole may be an appropriate strategy, however the failure to measure drug levels significantly limits the study’s impact and places patients at significant risk of resistance [139]. Current guidelines would advocate mandatory therapeutic drug monitoring to minimise the development of side effects and the development of resistance secondary to low plasma drug levels regardless of the azole of choice [29,132].

Evidence for the use of voriconazole for the treatment of CPA is supported by two prospective open multicentre trials and a review of case series, all of which involve small numbers of patients [74,140,141]. Overall response rates in these studies vary between 42.9% and 60.6%, with responses appearing to be greater in those with chronic necrotizing pulmonary aspergillosis (now known as SAIA) rather than chronic cavitary disease [74,140,141]. This highlights the importance of accurate stratification of patients in all future studies of antifungal efficacy. SAIA is a very specific subset of CPA, more akin to invasive aspergillosis, and its rapidly progressive nature and tissue invasion make it much more amenable to treatment. Similarly in those with chronic fibrosing pulmonary aspergillosis (CFPA), treatment aims to control and palliate rather than produce significant symptomatic improvement. Comparison of drug efficacy across the group as a whole potentially introduces significant bias to results, unfairly raising expectation. 

The use of posaconazole is currently reserved for those patients who have experienced side effects or progression on both itraconazole and voriconazole and the evidence base in CPA is limited. However response rates of 61% and 46% at 6 and 12 months respectively, suggest that posaconazole may have a role in the management of CPA [73].

Patient perceived improvement in their condition is also a key tool in assessing treatment success and long-term therapy azole has been linked to improved health status in patients with CPA [142,143]. Improvement rates of 47% and 50% at 6 and 12 months respectively demonstrate the impact these agents have on improving quality of life and patient experience and support the need to treat where possible [142]. However, these findings are tempered by the fact that a third of patients experienced deteriorating health status, highlighting the need for new and improved treatment strategies.

Isavuconazole, a newly discovered extended spectrum triazole, may also prove useful in the future management of CPA. Combining high levels of activity against yeasts, moulds and dimorphic fungi, predictable pharmacokinetics and excellent oral bioavailability with few serious side effects and fewer drug interactions compared to voriconazole therapy, this is an attractive new agent [144,145]. Moreover, potent fungicidal activity against both *A. fumigatus* and *A. flavus* has been demonstrated *in vitro* [144]. However, isavuconazole is currently only licensed for use in the treatment of invasive disease and efficacy within the CPA population has not been studied. Large population-based studies will be required to establish whether isavuconazole is non-inferior to established azole therapies in this group.

#### 5.1.2. Intravenous Therapy—Liposomal Amphotericin B and Echinocandins

Liposomal amphotericin B is used in CPA in instances of azole intolerance, failure of azole therapy or rapidly progressive disease, although the evidence base is very limited. Targeting the ergosterol within the fungal cell membrane, liposomal amphotericin alters cell permeability leading to cell lysis and death and has broad spectrum activity against *Aspergillus* spp. Response rates of 65% have been documented in CPA following administration of liposomal amphotericin B. However, the development of acute kidney injury in up to a third of patients makes amphotericin a difficult agent in what is often an elderly and multi-morbid population [10,90,146].

The echinocandins, micafungin and caspofungin, act through inhibition of β-(1,3)-d-glucan synthase, an enzyme that is necessary for the synthesis of essential β-(1,3)-d-glucan of the *Aspergillus* cell wall, leading to cell lysis through osmotic stress and fungal death. Like liposomal amphotericin B, they are used where there is azole intolerance, failure of azole therapy or rapidly progressive disease, although the evidence base is limited. Studies demonstrate the effective use of micafungin in the treatment of both CPA and SAIA, with clinical efficacy rates of up to 68.4% reported [147,148,149]. Non-inferiority to voriconazole, with a significantly reduced side effect profile, has also been demonstrated with micafungin over a four week period, although the perceived reduction in side effects should be viewed with caution as courses of voriconazole are often much longer in duration and very rarely given intravenously in standard treatment regimens [150].

Evidence for the use of caspofungin in CPA is also very limited, although non-inferiority to micafungin therapy has been demonstrated in a small, blinded randomised control trial [151]. Cyclical caspofungin therapy, with oral azole maintenance therapy, has also been demonstrated to confer some benefit in patients with sarcoidosis complicated by CPA [23]. The ideal duration of therapy for either drug is not clearly defined, although 3–4 week courses, repeated according to clinical need have been suggested [31]. Certainly courses of less than 2 weeks are not recommended and patients have been treated for up to three months with demonstrable clinical benefit [152]. Larger randomised control trials are required to establish the efficacy of both intravenous agents and their non-inferiority to alternative azole therapy. This will be difficult given their primary role as salvage therapy for the most difficult, and often unwell, patients. 

Expert opinion recommends the use of either liposomal amphotericin B or micafungin or caspofungin, in intermittent or continuous dosing regimens, for patients with multi- or pan-azole resistant *A. fumigatus* [152].

The widely differing efficacy rates demonstrated for both oral and intravenous antifungal agents represent the disparities in sample size, duration of therapy, drug dosing and therapeutic drug monitoring, and definition of treatment response used by different research groups. This highlights the needs for standardisation of treatment protocols and the identification of disease-specific markers representative of response to treatment. Subsequent trials will then provide a definitive insight into the best treatment regimens informing standards of care.

#### 5.1.3. Novel Antifungal Therapies

The development of new, ideally oral agents targeting *Aspergillus* spp., particularly *A. fumigatus*, remains critical to the delivery of high quality care to this patient group. Numerous new compounds are being explored as potential antifungal candidates with different, but as yet unidentified, drug targets from those of the currently licensed antifungal agents [153,154,155,156]. Exploration of the design and clinical utility of modified azoles is running in parallel to novel drug discovery in the hope that modification produces selective and specific targeting of fungal cytochrome P450 thus reducing issues surrounding drug interactions and host toxicity related to activation of host CYP3A4 [157]. Although drugs such as VT-1161 are currently in phase two trials, the yield from azole modification attempts remains low, with no modified azoles currently approaching availability for widespread clinical implementation [158].

### 5.2. Adjunctive Therapies

#### 5.2.1. Gamma Interferon Replacement Therapy

Given the emerging evidence that anomalies in cellular immunity, particularly deficiencies in the production and response to γ interferon, it is possible that replacement therapy may be a useful adjunct in those patients with CPA with demonstrable deficiency [35,36,37]. Clinical benefit from subcutaneous replacement therapy has been demonstrated in small case series and further work is required to establish the place of interferon γ in the treatment of CPA [10].

#### 5.2.2. Surgical Management of CPA

For patients with CPA, surgery has two key roles: the alleviation of disabling or life-threatening symptoms and, for a select group of patients, the potential for cure. Given that CPA affects only a small proportion of the population, many with cardiovascular and respiratory multi-morbidities that preclude surgery, the evidence surrounding surgical intervention is limited to large case series assembled over prolonged periods of time [133,159,160,161,162,163]. 

The most common operative intervention for patients with CPA is a lobectomy, with sequential lobectomies for bilateral disease being used in certain settings [133,159,161]. Other operative approaches are often undertaken with pneumonectomy, decortication, sublobar resection, segmentectomy, thoracoplasty, cavernostomy, bullectomy and pleurectomy all being documented [133,159,160,161,162,163]. Immediate post-operative complications are most common in those patients with CCPA, affecting up to 27% of individuals [133,161,162,163]. Complications in these patients largely mirror those seen for thoracic surgery as a whole, with the most common including bleeding, infection and the development of an empyema, post-operative respiratory failure and prolonged air leak with/without formation of a bronchopleural fistula [133,159,160,161,162,163].

Recent evidence has suggested that video-assisted thoracoscopy (VATS) may be a useful alternative to thoracotomy in selected patients, thus reducing morbidity and hospital stay [164,165]. This technique may be more suited to patients with simple aspergilloma requiring lobectomy alone. Pneumonectomy via VATS has not been demonstrated in this context and the limited evidence presented demonstrates both increased length of hospital stay and pleural drainage post-operatively in patients with CCPA when compared to simple aspergilloma [165]. Conversion to thoracotomy for adhesions, bleeding or hilar involvement was also highlighted in one series [164]. This demonstrates that careful case selection, operator experience and disease location are critical in determining the success of this approach. 

Thirty day mortality risk following surgery also varies between centres, from zero to a maximum of 4.3%, with this data reflecting largely open procedures [133,159,160,161]. The risk of both post-operative morbidity and mortality increases when resections are undertaken for anything other than simple aspergilloma [133,159,161,162]. Pneumonectomy, pre-operative massive haemoptysis, increasing age and weight loss have also been identified as factors predicting increased morbidity and mortality. Conversely female gender and a predicted forced expiratory volume in one second of >75% confer a positive prognosis [163,166,167]. Ten year survival rates post-operatively tend to be higher in the simple aspergilloma group, ranging from 62.5% to 92% compared to 68.5%–79.6% in those with complex disease [160,161,163]. In reality, the highly variable survival rates represent varying practices in surgical selection and the inclusion of deaths from other causes. Actuarial survival is often reported in these series due to inherent difficulties in identifying the extent to which CPA contributed to death, given the complex respiratory morbidity within this patient group.

The risk of recurrence post-operatively is difficult to quantify given the heterogeneous nature of the patients included within the surgical cohorts studied. Recent figures quote a recurrence rate of 26%, highest in those patients with CPA [133]. Recurrence can be classified as early or late, with early recurrence affecting the pleural space, usually resulting from surgical spillage of *Aspergillus* material intraoperatively, and the development of an empyema. In the event of recognised contamination, application of either 2% taurolidine or amphotericin B deoxycholate may reduce the risk of subsequent *Aspergillus* empyema, space infection and/or bronchopleural fistula, with taurolidine having demonstrated efficacy against all clinical isolates in one surgical series [29,133]. Three months of adjunctive antifungal therapy peri-operatively is recommended following surgical spillage [29,31]. Late recurrence is usually parenchymal in origin and no models currently exist to identify individuals most at risk. It is thought that a combination of genetic factors and pre-existing worsening lung disease are the most significant factors contributing to the development of recurrence in these patients although other factors, such as post-operative steroid exposure, may also be important [29,31]. Adjunctive post-operative antifungal therapy may play a role in preventing disease recurrence although it is not clear which subset of patients benefit the most from this approach [29,166,168]. Large, randomised, multi-centre trials are required to fully establish the role of adjuvant antifungal therapies post-operatively, a not insignificant undertaking.

#### 5.2.3. Haemoptysis and Bronchial Artery Embolisation

Haemoptysis occurs in patients with both chronic pulmonary aspergillosis and simple aspergilloma, secondary to neovascularization within the infected area. Vessels are derived from the systemic circulation, most commonly the bronchial arteries, although collaterals originating from the intercostal, subclavian and internal mammary arteries are also described [29]. For patients with mild or moderate haemoptysis, management is often conservative, with pharmacological anti-fibrinolytic therapy, for example tranexamic acid, often proving successful [29]. For patients with severe or life threatening haemoptysis, bronchial artery embolization proves a useful temporising measure, particularly in those where surgery is precluded due to associated respiratory morbidity. With the technical success of the procedure being rated at between 50% and 90%, embolization can be a truly lifesaving intervention, although the effects are often temporary and the procedure itself carries not insignificant risks of both cerebral and spinal stroke [29,132,169,170].

The installation of amphotericin B directly into the affected cavity has also been used in a small number of patients in an attempt to suppress massive haemoptysis [171,172,173]. Success has been reported in a significant number of individuals, with haemostasis achieved at between 24 h and 7 days [171,172,173]. Although an attractive alternative in those where embolization or surgery is not possible, this procedure is not without risk, and respiratory co-morbidity may preclude successful placement of a catheter, as may severity of the bleed.

## 6. Treatments Barriers

### 6.1. Side Effects

The side effect profile of long-term azole therapy is poorly described in this patient population. All three azole therapies have a significant number of potential side effects with neuropathy and hepatotoxicity spanning all three agents. Hepatotoxicity is usually reversible and has been reported with itraconazole, voriconazole and posaconazole. For itraconazole, hepatotoxicity has been reported in 7% of individuals, with significantly higher rates seen for voriconazole in both invasive and chronic aspergillosis, 14.6% and 17.6%, respectively [85,174,175]. This supports data from the FDA adverse event reporting system; although it should be remembered this data does not take into account the underlying treated condition or patient comorbidities [176]. Data for posaconazole is limited; low rates of hepatotoxicity have been documented in the treatment or prophylaxis of invasive disease, with no hepatotoxicity reported in a retrospective cohort analysis of patients with CPA [73,177]. There appears to be lack of cross hepatotoxicity within the azole group, allowing affected individuals to be successfully, albeit cautiously, re-challenged with a second agent [73,178,179].

All azole therapies carry the potential risk of peripheral neuropathy. Baxter *et al.* assessed prevalence of neuropathy in a cohort of CPA patents receiving long-term therapy [180]. Peripheral neuropathy affected 10% of patients with a median time to onset of 3 months. Itraconazole neuropathy was demonstrated in 17% of treated patients, with figures of 9 and 3% for voriconazole and posaconazole, respectively. Most neuropathies associated with azole therapy are predominantly sensory in nature although bilateral lower limb weakness and quadriparesis have also been reported [180,181,182,183]. Happily for the majority, neuropathy resolves on cessation of therapy although some individuals experience irreversible damage to both small and large nerve fibres with ongoing symptoms despite drug cessation [180,181,183]. The mechanism of azole induced neurotoxicity is, at present, unknown making prevention, or identification of susceptible individuals, difficult.

Key specific side effects for both itraconazole and voriconazole are discussed below.

#### 6.1.1. Itraconazole

Itraconazole use has been linked to congestive cardiac failure although this remains a controversial topic in clinical practice. Although reported widely, cases are often confounded by pre-existing cardiac disease, multi-morbidity and polypharmacy, making causality difficult to assign [184,185,186,187,188]. In the main, cardiac function is reported to recover to baseline on cessation of the drug, with complete amelioration of symptoms [184,188]. Thought to be negatively ionotropic, the exact mechanism of itraconazole-induced cardiac failure remains to be elicited. At present, those thought to be at greatest risk of cardiac failure are patients with known cardiac impairment, although the observation of left ventricular dysfunction in those with structurally and functionally normal hearts confounds this and makes identification of susceptible individuals difficult. Hypertension, ventricular ectopics and arrhythmia have also been very rarely reported.

#### 6.1.2. Voriconazole

Cutaneous side effects are estimated to occur in less than 10% of patients on voriconazole therapy and take many forms, although the underlying mechanism remains unknown [189,190]. Severe photosensitivity is perhaps the most feared complication, but forms only part of a spectrum of conditions representing accelerated sun-induced skin changes including photoaging, actinic keratosis and skin cancers [191]. These risks can be, in part, negated by the use of high factor sunscreen; however compliance with this in the long term is likely to be poor. Multiple other manifestations are described including chelitis, discoid lupus, Stevens-Johnsons syndrome and toxic epidermal necrolysis [189,192].

Drug induced periostitis has also been reported in a number of individuals receiving long term voriconazole therapy, albeit in different patient populations [193,194,195,196,197]. The mechanism for this is poorly understood although fluoride accumulation has been implicated [194,195,198]. This is a plausible theory given that voriconazole is rich in fluoride, fluoride is known to increase osteoblastic stimulation leading to abnormal bone structure, and elevated levels have been demonstrated in affected patients [194,195,199]. Conversely, recent *in vitro* work suggests fluoride-independent pathways are responsible for periostitis, contradicting current literature. Further well designed *in vivo* studies are required for corroboration [200].

Transient visual side effects are unique to voriconazole and are identified in up to 30% of patients [201,202]. Changes experienced are wide-ranging and include altered light perception, photophobia and visual hallucinations but are largely transient and do not adversely affect visual acuity [201,202,203,204]. Occurring as early as 30 minutes post-administration, the mechanism of these changes is largely unknown although recent research has identified drug blockade of TRPM1 and TRPM3 channels within the retinal epithelium and brain as causal [201].

In addition to the key side effects listed above, all azoles have the propensity to cause a myriad of other side effects, which reduce patient tolerability and clinical application. In combination with resistance, the side effect profiles of the commonly used azoles are a key limiting factor in the treatment of CPA. There is a significant, and as yet unmet, need for an increased pool of antifungal agents with a reduced side effect profile which will increase compliance and in turn stem developing azole resistance and the need for intravenous antifungal therapy.

### 6.2. Drug Interactions

Multiple drug interactions are documented for all azole therapies, making the introduction of azole therapy challenging [205]. Administration of azole therapy with drugs that induce the enzyme cytochrome P450 results in a significant reduction in blood levels of the administered azole. Subsequently it is almost impossible to achieve therapeutic drug levels, leading to both poor clinical outcome and the risk of establishing new fungal resistance mechanisms. In HIV-infected individuals, there is uncertainty about dual dosing of efavirenz (and other similar agents) and itraconazole, although it is likely a larger itraconazole dose is required to combat enzyme induction, and combined therapy with voriconazole/efavirenz is not recommended. Conversely both posaconazole and itraconazole are potent inhibitors of CYP-3A4, which precludes co-administration of either of these agents with drugs metabolised using this pathway. Failure to identify potential drug interactions in this situation leads to increased serum levels of the non-azole agent, increasing the risk of side effects. Voriconazole doesn’t appear to inhibit CYP-3A4 to the same degree, instead demonstrating profound inhibition of CYP-2C9 and CYP-2C19 producing a different spectrum of drug interactions.

Co-existent respiratory pathology also raises significant treatment dilemmas with NTM infection providing one of the biggest challenges. With rifampicin or rifabutin forming the backbone of most NTM treatment regimens, hepatic induction of CYP-450 and increased azole metabolism make therapeutic drug levels a virtual impossibility. Persistently sub-therapeutic itraconazole levels have been demonstrated in both the presence of rifampicin and rifabutin and, although not formally studied, the effect is likely to be similar in patients on both voriconazole and posaconazole [206]. Although therapeutic voriconazole levels have been reported in the context of co-existent *Aspergillus* and *M. xenopi* infection, concomitant dosing is fraught will difficulty and risks the development of resistance in the fungus [207]. For these patients, individual decisions will need to be made about the dominant disease and risk of progression, with individual treatment regimens applied sequentially rather than concurrently.

### 6.3. Drug Resistance in A. fumigatus—An Emerging Problem

The development of drug resistance, or the acquisition of a primarily resistant strain, is often catastrophic for the individuals involved and, given the limited pool of therapeutic options available, forms a major barrier to long term therapy. The mechanisms driving the development of azole resistance in *A. fumigatus* are not well understood. It is unclear whether clinically significant azole resistance is acquired *de novo* within the host due to the selection pressure of prolonged azole therapy, or through host inhalation of an azole resistant environmental isolate, resistance developed in the context of prolonged exposure to agricultural azole based fungicides [95,208,209,210,211,212,213,214].

Itraconazole resistance was first reported in the CPA population in 1997 but was initially the focus of limited research [215]. The publication of two major epidemiological studies demonstrating the importance of emerging azole resistance in the UK and the Netherlands changed this, acting as a catalyst for further epidemiological study and resistance surveillance [95,216]. As a direct consequence of this, the current global prevalence of azole resistance is estimated at between 0.3% and 28% [217,218,219,220,221,222]. With increasing agricultural and clinical use this figure is likely to increase further with the rapid development of resistance demonstrated against newly released antifungal therapies. For example resistance to isavuconazole, initially thought to retain clinical efficacy against resistant strains, is already being demonstrated. This confirms the need for continued vigilance, routine testing of all isolates to allow early identification of resistance and antifungal stewardship if azole therapy is to retain a long term role in the treatment of this disease [223,224,225,226]. 

Resistance is probably under-appreciated because of the low culture yield and even with culture positivity; effective therapy can be impeded by unnecessary pre-emptive or empiric therapy or delayed by susceptibility testing [96,98]. Two novel methods for direct detection of resistance, now commercially available, have demonstrated good results although the diversity of resistance mechanisms and associated genetic variability confer significant challenges to demonstrating adequate assay sensitivity and specificity in the context of CPA samples [227,228,229].

### 6.4. Other Barriers to Treatment of CPA

Perhaps an even greater barrier to treatment than both the developing and evolving resistance profiles of *A. fumigatus*, and the not insignificant side effect profiles of the current treatment modalities, is a lack of education, awareness and resources for treatment of these patients in both the developed and developing world. The Global Action Fund for Fungal Infections *(GAFFI)* estimates CPA affects over 3 million people worldwide and multiple studies using literature searches and population modelling have identified CPA as a truly global problem spanning continents and differing disease entities [2,7,8,21,230,231,232,233,234,235]. Much of the population modelling described has been undertaken in the developing world where economies and health infrastructure are poorly developed and the ability to recognise and treat the disease early is limited. From a health economics point of view it could be argued that these countries, which are often also plagued by other serious fungal infections, have the most to gain from intensive education of politicians, clinicians and scientists. However the situation in the developed nations is far from perfect. Recognition of CPA is limited by poor awareness of medical mycology by both clinicians and research scientists. In some of the most developed health care systems in the world it is likely that many patients remain undiagnosed, experience a delay in diagnosis or delay in institution of appropriate treatment, conferring significant additional morbidity and mortality. For each and every one of these patients, in each and every country, this is a tragedy, which will only be addressed through education, improved awareness and equality in access to healthcare.

## 7. Long Term Management Goals

Management of CPA is a long-term undertaking with treatment being prolonged, often over a period of years. Cure is not possible for the vast majority of these patients, so treatment aims to stabilise disease, prevent progression and reduce haemoptysis and fibrosis. The introduction of therapy within the CPA population does not guarantee either fast or definitive improvement. Response to treatment is slow although response, or failure to respond, can usually be determined following 6 months of therapy. After completion of 9 months of therapy almost all patients who are destined to respond will have done so [73]. Response to treatment can be difficult to assess although a combination of serological, radiological and clinical measures are used [29]. A fall in *Aspergillus* specific IgG is often used as a surrogate marker, however the evidence base for this is extremely limited and a very poor serological response can be mounted in certain individuals despite extensive disease. At the present time only radiological changes, specifically reduction in cavity wall or pleural thickness demonstrated on serial CT scanning, provide a degree of objectivity in defining response to therapy highlighting the importance of long term imaging surveillance in this patient group [29,76].

The need for long-term therapy is supported by the demonstration of high relapse rates following cessation of antifungal therapy, with rates of up to 30% quoted [75,236]. Phenotypic associations with relapse include younger age, prolonged therapy with antifungals, slow radiological resolution and multi-lobar disease [236]. For patients with stable disease who do not relapse it could be argued that continued therapy is beneficial to prevent insidious and continuing damage.

Despite therapy, mortality from CPA is often very high, with rates of 27% reported at 30 months, rising to 50% at five years [13,237]. The fact that significant respiratory co-morbidity is often associated with CPA means that death in these individuals may not be solely due *Aspergillus*, however better treatment strategies would undoubtedly reduce mortality on a global scale.

## 8. Conclusions

CPA is a multifaceted disease that requires further research and investment in almost every area. Early recognition is hampered by non-specific symptoms/radiology and poor awareness, with the diagnostic modalities available not yet fully established for assessment of the target population. Appreciation of those individuals most at risk remains limited and further exploration of genetic and immune risk factors is badly needed. Treatment is limited by the availability of a small pool of often poorly tolerated oral antifungal therapies and emerging resistance is limiting the utility of these. The lack of defined endpoints for monitoring improvement or progression of established disease proves problematic both clinically and scientifically as drug development relies on such markers to bring novel agents to clinical trials. Further investment and collaboration between clinicians, scientists and the pharmaceutical industry globally is required to address these issues and raise the profile of the disease.

## Figures and Tables

**Table 1:** Conditions predisposing to SAIA [17,31,51,52,53,54].

Conditions Predisposing to SAIA
Diabetes mellitus
Malnutrition
Alcohol excess
Advancing age
Prolonged use of oral corticosteroids
Administration of immunosuppressive therapy e.g., In the treatment of connective tissue disease, post solid organ transplantation.
COPD
Radiotherapy
Nontuberculous mycobacterial infection
HIV infection

**Table 2:** Mandatory diagnostic tests for patients suspected of having CPA.

Immunology/Serology	Sputum Microbiology	Radiology
*Aspergillus* IgG/precipitins	Microscopy	CXR
Immunoglobulins and electrophoresis	Culture (including fungal culture)	
Functional antibody testing (*Tetanus*, *Haemophilus*, *Pneumococcus*)	Sensitivity (including resistance testing of any isolated *Aspergillus* spp.)	
Mannose binding lectin levels	Sputum *Aspergillus* PCR	CT thorax

**Table 3:** Differential diagnosis of CPA. * Commonly co-existent with CPA.

	Differential Diagnosis
Malignancy	Lung cancer, pulmonary metastases
Vasculitis	Particularly granulomatosis with polyangiitis
Pulmonary infarction	For example following large pulmonary embolism
Post radiotherapy change	Extensive radiotherapy field often produce fibrotic change that can mimic CFPA
Mycobacterial infection *	*M. tuberculosis*, Nontuberculous mycobacteria
Fungal infection	Chronic cavitary pulmonary histoplasmosis, paracoccidioidomycosis and coccidioidomycosis
Bacterial infection *	Necrotizing pneumonia

**Table 4:** Differential diagnoses for a patient with a positive *Aspergillus* IgG.

Differential Diagnosis of a Positive Aspergillus IgG
Asymptomatic individual
Aspergillus bronchitis
Acute invasive aspergillosis
Subacute invasive aspergillosis
Chronic pulmonary aspergillosis
Allergic Bronchopulmonary aspergillosis/fungal sensitization
Recent primary community acquired pulmonary aspergillosis

## Abbreviations

The following abbreviations are used in this manuscript:

ABPA	allergic bronchopulmonary aspergillosis
CPA	chronic pulmonary aspergillosis
CCPA	chronic cavitary pulmonary aspergillosis
CFPA	chronic fibrosing pulmonary aspergillosis
CNPA	chronic necrotizing pulmonary aspergillosis
COPD	chronic obstructive pulmonary disease
CT	computed tomography
CXR	chest X-ray
CRP	C-reactive protein
EBUS	endobronchial ultrasound
ESCMID	European Society of Clinical Microbiology and Infectious Diseases
ESR	erythrocyte sedimentation rate
ERS	European Respiratory Society
GAFFI	Global Action for Fungal Infections
GM	galactomannan
IDSA	Infectious Diseases Society of America
LFD	lateral flow device
MALDI-TOF	matrix-assisted laser desorption/ionization time-of-flight
MIC	minimum inhibitory concentration
NTM	nontuberculous mycobacteria
ODI	optical density index
PET	positron emission tomography
POC	point of care
PCR	polymerase chain reaction
PV	plasma viscosity
SAIA	subacute invasive aspergillosis
SNP	single nucleotide point mutation
TB	tuberculosis
